# Segregation over time in functional networks in prefrontal cortex for individuals suffering from pathological fatigue after traumatic brain injury

**DOI:** 10.3389/fnins.2022.972720

**Published:** 2022-09-08

**Authors:** Simon Skau, Birgitta Johansson, Hans-Georg Kuhn, William Hedley Thompson

**Affiliations:** ^1^Institute of Neuroscience and Physiology, Sahlgrenska Academy, University of Gothenburg, Gothenburg, Sweden; ^2^Department of Pedagogical, Curricular and Professional Studies, Faculty of Education, University of Gothenburg, Gothenburg, Sweden; ^3^Department of Applied Information Technology, University of Gothenburg, Gothenburg, Sweden; ^4^Department of Clinical Neuroscience, Karolinska Institutet, Stockholm, Sweden

**Keywords:** connectivity, modularity, fNIRS, pathological fatigue, fatigability, state fatigue

## Abstract

Pathological fatigue is present when fatigue is perceived to continually interfere with everyday life. Pathological fatigue has been linked with a dysfunction in the cortico-striatal-thalamic circuits. Previous studies have investigated measures of functional connectivity, such as modularity to quantify levels of segregation. However, previous results have shown both increases and decreases in segregation for pathological fatigue. There are multiple factors why previous studies might have differing results, including: (i) Does the functional connectivity of patients with pathological fatigue display more segregation or integration compared to healthy controls? (ii) Do network properties differ depending on whether patients with pathological fatigue perform a task compared to periods of rest? (iii) Are the brain networks of patients with pathological fatigue and healthy controls differently affected by prolonged cognitive activity? We recruited individuals suffering from pathological fatigue after mild traumatic brain injury (*n* = 20) and age-matched healthy controls (*n* = 20) to perform cognitive tasks for 2.5 h. We used functional near-infrared spectroscopy (fNIRS) to assess hemodynamic changes in the frontal cortex. The participants had a resting state session before and after the cognitive test session. Cognitive testing included the Digit Symbol Coding test at the beginning and the end of the procedure to measure processing speed. We conducted an exploratory network analysis on these resting state and Digit Symbol Coding sessions with no *a priori* hypothesis relating to how patients and controls differ in their functional networks since previous research has found results in both directions. Our result showed a Group vs. Time interaction (*p* = 0.026, *η_*p*_*^2^ = 0.137), with a *post hoc* test revealing that the TBI patients developed higher modularity toward the end of the cognitive test session. This work helps to identify how functional networks differ under pathological fatigue compared to healthy controls. Further, it shows how the functional networks dynamically change over time as the patient performs tasks over a time scale that affect their fatigue level.

## Introduction

*Pathological fatigue* is when the general tendency of *fatigability* and the *sensation of fatigue* is perceived to interfere with everyday life (Skau et al., 2021). Pathological fatigue is often a consequence of trauma to, or disturbance in, the central nervous system (Johansson and Rönnbäck, 2014; Berginström, 2019). The prevalence of pathological fatigue is estimated to be between 36–77% after stroke, 45–73% after traumatic brain injury (TBI), 38–83% in multiple sclerosis (MS), and 28–58% in Parkinson’s disease (Kluger et al., 2013). It is also associated with conditions, such as exhaustion disorder (Sandstrom et al., 2005; Krabbe et al., 2017), infection of the central nervous system (Morris et al., 2015), or hormonal imbalance (Möller et al., 2014), together with additional symptoms such as sensitivity to light and sound and irritability. Individuals suffering from pathological fatigue after mild TBI often report an increased sensation of fatigue after mental activity with an abnormally long recovery time (Johansson and Ronnback, 2017). Studies of pathological fatigue after moderate to severe TBI using functional magnetic resonance imaging (fMRI) indicate a dysfunction within cortico-striatal-thalamic circuits (Kohl et al., 2009; Nordin et al., 2016; Berginstrom et al., 2017; Möller et al., 2017; Wylie et al., 2017).

**FIGURE 1:**
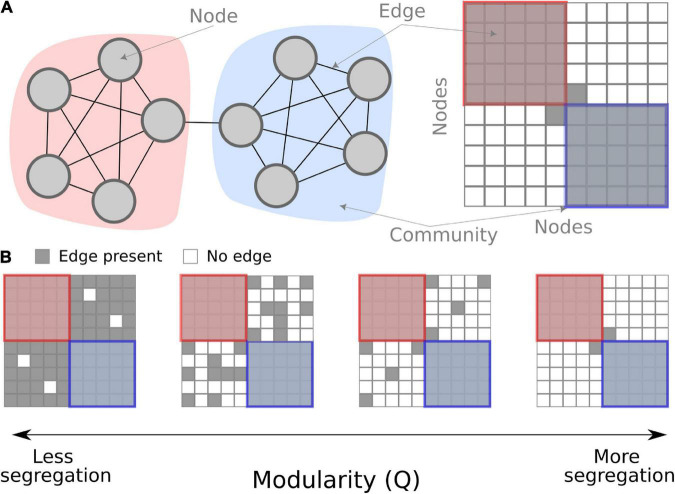
Conceptual overview of modularity in a network. **(A)** A schematic network of 10 nodes in two communities (red and blue) connected by binary edges. On the left, the nodes are shown graphically, and on the right, the same schematic network is shown as a connectivity matrix. **(B)** Examples of modularity as a measure. When there are fewer between-community edges, the modularity measure is higher, interpreted as more segregation.

The interplay between integration and segregation within brain networks is considered a critical property of brain function and cognition (Sporns, 2013). Among the many network measures, modularity is one of the more commonly used when studying fatigue. It is a global measure that quantifies the segregation of the entire network. Based on the co-variation of functional brain activity among different brain regions, groups of nodes get clustered together into communities (note, in network theory, these are called “communities,” which is analogous to “brain networks” or “resting-state networks” often used in cognitive neuroscience). Modularity quantifies how tight-knit these communities are compared to chance. Modularity is high if there are fewer between-community connections (see Figure 1) which are interpreted as higher segregation between communities. Contrarily, low modularity is indicative of either low segregation or high integration in the network.

Numerous neuroimaging studies on patient populations with fatigue have used functional connectivity measures, but the findings are inconclusive. Some studies have found increased integration or connectivity in pathological fatigue. Messé et al. (2013) investigated network properties for TBI patients, with and without post-concussion symptoms (including fatigue). The group with post-concussion symptoms had lower modularity, i.e., a less segregated functional network. Similarly, higher self-reported fatigue in chronic fatigue patients was associated with a lower degree of connectivity for the medial frontal cortex with the rest of the brain (Gay et al., 2016). On the other hand, Høgestøl et al. (2019) found that connectivity in the default modal network increased for MS patients with high severity of depressive and fatigue symptoms. In contrast, Kim et al. (2015) reported that patients with chronic fatigue syndrome displayed a decrease in global efficiency, a measure for network integration.

In cohorts with healthy adults, previous research has found that inducing fatigue results in an decrease in segregation measured by decreased modularity (Ben Simon et al., 2017) and an increase in path length, another measure of segregation (Sun et al., 2014). Wylie et al. (2020) identified a network made up of the dorsolateral prefrontal cortex (DLPFC), ventromedial PFC (VMPFC), dorsal anterior cingulate cortex (dACC), anterior insula, and the striatum, which showed less connectivity when state fatigue increased after cognitive activity.

In sum, researchers have linked changes in connectivity, through common topographical measures such as modularity, to pathological fatigue and cognitive fatigability in healthy adults. However, whether there is a change in integration or segregation is unclear. Further, we do not know if different factors in design and population impact the varying results. For example, it is unclear if cognitive fatigability (i.e., the decrement in cognitive performance over a consecutive time) affects the modularity of networks differently for healthy adults compared to individuals suffering from pathological fatigue. Moreover, study design aspects, such as the duration of cognitive activity for inducing cognitive fatigue, can vary [e.g., 20 min in Sun et al. (2014), but 2.5 h in Skau et al. (2019)]. Further, some studies above performed their network calculation from resting-state sessions, while others had participants perform a task. In summary, the following three factors relating to network theory and fatigue are still not fully understood:

1. Do the brain networks of patients with pathological fatigue display more segregation or integration compared to healthy controls?
2. Do network properties differ depending on whether patients with pathological fatigue perform a task compared to periods of rest?
3. Are the brain networks of patients with pathological fatigue and healthy control differently affected by prolonged cognitive activity?

**FIGURE 2:**
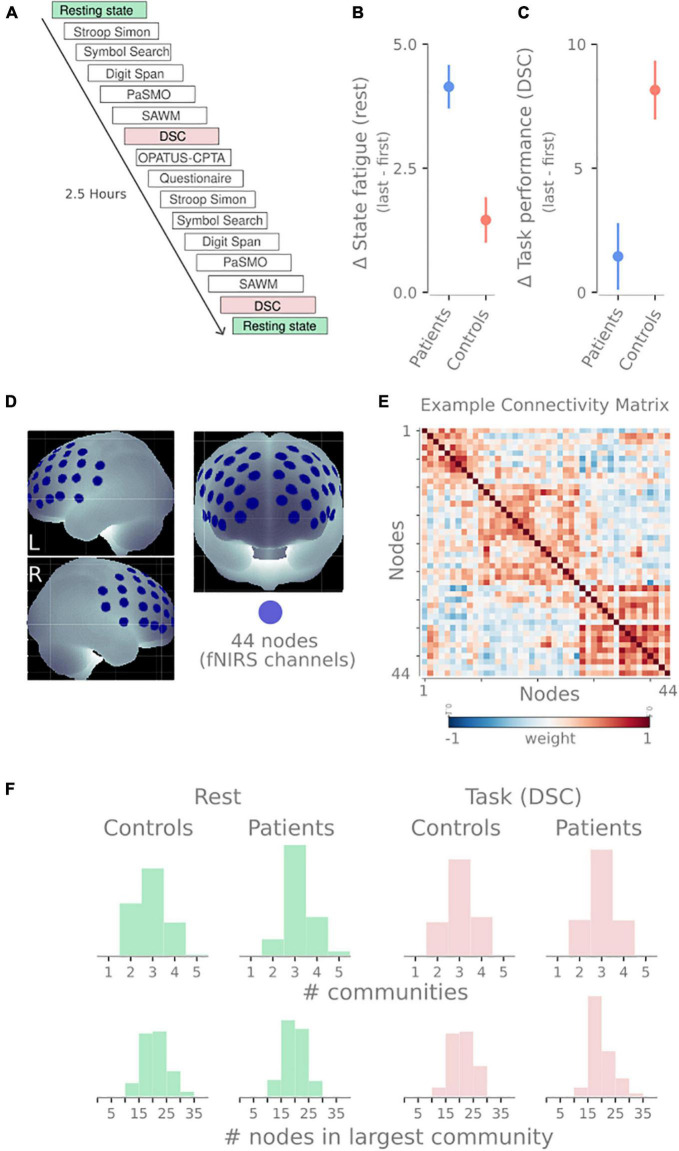
Overview of design and methodology. **(A)** Timeline of tasks performed by participants in this study. The blocks analyzed in this manuscript have been highlighted: resting-state sessions (green) and digit symbol coding tasks (DSC, red). **(B)** The difference in self-reported state fatigue following the resting-state session (last first) for both groups. **(C)** Difference in task performance in the DSC task (last-first). Panels **(C,D)** show data previously reported in Skau et al. (2019). **(D)** The 44 recording sites on the frontal cortex. These 44 recording sites become nodes in the network. **(E)** An example connectivity matrix from one resting-state session showing three communities for the 44 nodes depicted in panel **(B)**. Similar to the connectivity matrix in 1A, but with weighted edges instead of binary edges. **(F)** Descriptive statistics of the community detection properties. Histograms show the number of communities (top) and the number of nodes in the largest community (bottom) for rest (green) and DSC task (red) and both patients and healthy controls.

In this study, we provide evidence relating to each of these questions. We use the functional near-infrared spectroscopy (fNIRS) data of pathological fatigue after mild TBI from Skau et al. (2019). fNIRS is an optical imaging technique that applies near-infrared light to measure the change in oxygenated and deoxygenated hemoglobin a couple of centimeters down into the neocortex. Twenty individuals suffering from pathological fatigue after mild TBI and twenty healthy controls performed cognitive tests for about 2.5 h. The test battery consisted of 6 neuropsychological tests done twice, intermediated with a sustained attention task. Throughout the experiment, multiple resting-state sessions were done (see Figure 2A).

We conducted an exploratory network analysis with no *a priori* hypothesis related to how patients and controls differ in their functional networks considering previous articles have found results in both directions. We chose modularity to evaluate segregation since modularity is an intuitive, single global measure. Previous studies have used it, and it circumnavigates specific problems relating to network measures over time (see Thompson et al., 2020). We analyze the contrast in modularity before and after performing a long battery of tests in healthy adults and patients. Further, we analyze the first and last resting-state session and the Digit Symbol Coding (DSC) that measures processing speed. This work helps identify how network analyses of pathological fatigue differ from healthy controls and dynamically change over time as the patient performs tasks over a time scale that affects their fatigue.

## Materials and methods

### Study participants and protocol

Details of the protocol and descriptions of the cognitive and neuropsychological tests included in the test session but not analyzed here are presented in Skau et al. (2019).

Twenty individuals with pathological fatigue after mild TBI (minimum 5 months after injury) were recruited from the Department of Neurology, Sahlgrenska University Hospital, Gothenburg. Inclusion criteria were as follows: diagnosed with mild TBI according to the definition proposed by The World Health Organization Collaborating Center for Neurotrauma Task Force (Carroll et al., 2004); scoring above the cut-off score of 10.5 on the Mental Fatigue Scale (MFS) (Johansson and Rönnbäck, 2014); aged 20–65 years and not suffering from any other psychiatric or neurological disorders. All participants recovered well and were independent in their daily living, except for their pathological fatigue. Six individuals received methylphenidate drug treatment but suspended the treatment 1 week before the assessment. As reported in Skau et al. (2019), no significant differences concerning the cognitive test results and ratings on MFS were detected between these six individuals compared to the other individuals with TBI. Twenty-one healthy controls who neither suffered from pathological fatigue (below 10.5 points on MFS) nor had any psychiatric or neurological disorders were recruited from the general community at request. One control subject was excluded due to failure to follow instructions. The Regional Ethical Review Board in Gothenburg approved the study (reference number: 028-16). The participants gave their informed written consent before the assessment and were told they could withdraw at any time.

### Experimental design

Each participant was seated in a chair next to a table with a computer screen. All tests were performed sitting in the same location. Depending on the task requirements, different responses from the participants, such as computer input (*via* mouse, tablet, or game controller), pen and paper, or verbal responses, were needed. The fNIRS cap with optodes attached was carefully placed on the participant’s head and worn throughout the experimental session. In order to minimize ambient light reaching the optodes at the scalp, the fNIRS cap was covered by another stretchable cap. The experiment consisted of two identical test sessions with six individual tests performed in the same sequence (Figure 2A). The two sessions were separated by a sustained-attention test with an 8-min one-back task (OPATUS-CPTA) and completing the MFS (Figure 2A). In total, the test procedure took 2 1/2 h. Participants were allowed to take a short break where they could drink water or stand up and stretch their legs between tests while keeping the fNIRS cap on.

Before and after the experimental procedure, participants rated their energy level on a visual analog scale (VAS). The VAS scale was a continuous line (10 cm) between the two end-points: “full of energy” and “totally exhausted, no energy left,” and was used to evaluate state fatigue. Mean and SD were 3.13 ± 2.0 and 7.27 ± 1.7 (for the patients) and 2.66 ± 1.5 and 4.12 ± 1.6 (for the controls) for the first and second VAS, respectively (see Figure 2B).

There were five separate occasions of 1-min resting-state recordings where participants were asked to focus on a fixation cross. These sessions were positioned: before the first task, after the first Stroop-Simon test, before the second Stroop-Simon test, after the second Stroop-Simon test, and right at the end of the experiment (see Figure 2A).

Digit Symbol Coding (DSC) is a subtest within the Processing Speed Index in WAIS-IV (Wechsler, 2010) that was used to measure attention, mental and psychomotor operation speed, and visual discrimination. Participants are asked to perform as many symbols as possible for 2 min. The raw score is the number of correct symbols performed. Mean and SD were 65.6 ± 11.7 and 67.0 ± 15.6 (for the patients) and 72.2 ± 10.9 and 80.4 ± 12.4 (for the controls) for the first and second test, respectively (see Figure 2C).

### Functional near-infrared spectroscopy data acquisition

The fNIRS measurements were performed using a continuous wave system (NTS) Optical Imaging System, Gowerlabs Ltd., United Kingdom (Everdell et al., 2005), using two wavelengths (780 and 850 nm) to measure changes in the concentration of oxygenated hemoglobin (oxy-Hb), deoxygenated hemoglobin (deoxy-Hb), and their total sum hemoglobin (tot-Hb). The system has 16 dual-wavelength sources and 16 detectors. The array consisted of 44 standard fNIRS channels (i.e., source/detector pairs) with a source-detector distance of 30, plus two short-separation channels with a source-detector distance of 10 mm, as suggested in previous studies (Gagnon et al., 2011; Brigadoi and Cooper, 2015). Short separation channels are only sensitive to hemodynamics in the scalp. Since the regular separation channels measure signals originating in both the brain and the scalp, the use of short-separation channels allowed us to regress the scalp signal from regular-separation signals to improve the brain specificity of the fNIRS measurement (Gagnon et al., 2011; Brigadoi and Cooper, 2015). The placement of the optodes was designed to encompass the frontal cortex, previously reported to be involved in executive function and cognitive control tasks (see Figure 2D; Roberts and Hall, 2008). Data were acquired at a sampling frequency of 10 Hz.

### Functional near-infrared spectroscopy data analysis

The fNIRS data were preprocessed using MATLAB (2018) and the MATLAB-based fNIRS-processing package *HomER2* (Huppert et al., 2009). The processing pipeline started with pruning the raw data such that channels were rejected if their mean intensity was below the instrument’s noise floor (1e-4 A.U.). The raw data was then converted to optical density. A high band-pass filter of 0.05 was used to correct for drift and a low band-pass 0.5 filter to remove pulse and respiration. The HomER2 functions *hmrMotionArtifactByChannel* and *hmrMotionCorrectSpline* were used to correct for motion artifacts. Optical density was converted to hemoglobin concentration with *hmrOD2Conc* with a default pathlength factor of 6.0 for both wavelengths. Before *hmrBlockAvg* was used, activity from short separation channels was regressed out of the long 44 standard channels. The short channel selected for regression was the one with the highest correlation to the respective long channel.

### Functional connectivity and network analysis

To create the networks, we used the 44 fNIRS channels as nodes in the network. To create the edges between the nodes, the preprocessed and denoised time series of each node were correlated with each other using Pearson correlations. This process creates a 44 × 44 symmetrical weighted connectivity matrix for each subject and session, representing the functional connectivity for that session (Figure 2E).

Before the community detection, the negative edges were set to 0. The Louvain community detection algorithm was used, as implemented in the *python-louvain* package (V0.15). Through the community detection algorithm, nodes are clustered into non-overlapping communities. The modularity of the network was calculated after the community detection. As there is stochasticity within the Louvain algorithm, it was run 100 times with the modularity calculated each time and the average modularity over all runs was used. The resolution parameter was set to 1, but to demonstrate that this parameter has not induced or influenced the results, Supplementary Figure 1 shows that this parameter has little effect on the results when jittering between 0.8 and 1.2.

One task or group could have varying community profiles leading to problematic comparisons (e.g., if every node is placed in a singleton community or all nodes belong to the same community). To illustrate that this was not the case, Figure 2F shows distributions of the number of communities detected and the size of the largest community. While there is a slight skewness difference between patients and controls at rest regarding the number of communities, they both have the same median (3). None of the distributions display extreme values rendering modularity comparison problematic. We included the number of nodes in the largest community to demonstrate that the community sizes were not the majority of nodes followed by 1–2 singleton communities.

### Statistics

We used the open-source program *JASP* version 0.13.1 for statistical analysis (Marsman and Wagenmakers, 2017). We conducted a repeated ANOVA with one between-group variable *Group* (TBI, controls), and two within-group variables *Time* (first, last) and *Activity* (rest, task), with *Age* as a covariate. *Post hoc t*-tests were performed with the Holm method used for multiple comparison correction. The datasets generated in the current study are available from the corresponding author on reasonable request.

Pearson’s correlations were used to evaluate if the change in state fatigue was linearly associated with the change in task performance and change in rest and task modularity. Delta scores (last–first) were used for the self-reported state fatigue measure (the VAS) and the delta DSC task performance, delta rest modularity, and delta task modularity.

## Results

**FIGURE 3:**
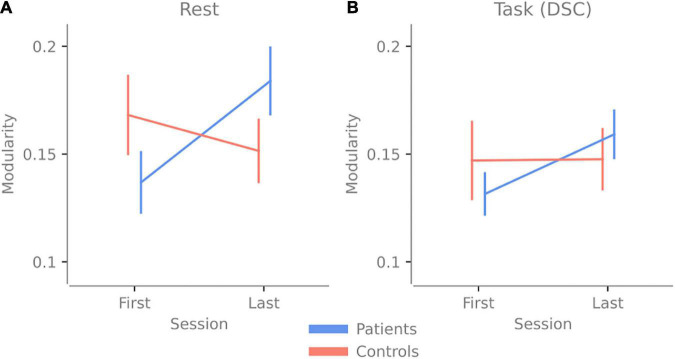
Change in modularity over 2.5 h of cognitive activity. Panel **(A)** shows the change in modularity between the first and last resting-state session. **(B)** The change in modularity during the first and last Digit Symbol Coding (DSC) task was done at the experiment’s beginning and end. Error bars indicate the standard error of the mean.

When analyzing the modularity scores, the repeated three-way ANOVA revealed a significant *Group* vs. *Time* interaction [*F*(1,34) = 5.399, *p* = 0.026 *η_*p*_*^2^ = 0.137]. *Post hoc* test showed higher modularity in TBI last > TBI first with *t*(19) = −2.812, with a Holms corrected *p*-value of 0.049 and a Cohen’s *d* of −0.653. This result suggests that no matter the activity (rest or task), patients have higher modularity after 2.5 h of cognitive activity (see Figure 3 and Supplementary Table 1 for the other *post hoc* results).

Since there was a Group vs. Time interaction, the main effect results of Group and Time need to be interpreted accordingly. There were no main effect differences for Group [*F*(1,34) = 0.127, *p* = 0.742 with a *η_*p*_*^2^ = 0.004], indicating no overall difference in modularity between patients and control when both time points are pooled together. There were no main effect differences for *Time* [*F*(1,34) = 1.054, *p* = 0.312 with a *η_*p*_*^2^ = 0.03], indicating that overall (when both groups are pooled together) there was no difference over time, even though the *post hoc* test showed that the TBI group had significantly higher modularity. There were no main effect differences for *Activity* [*F*(1,34) = 0.005, *p* = 0.947 with a *η_*p*_*^2^ = 1.337e-4], indicating that overall, for both groups, there was no difference in modularity between rest and task.

Finally, delta VAS (the changes in self-reported state fatigue) correlated negatively with the DSC performance delta score (*r* = −0.518, *p* < 0.001), indicating that the more change in delta VAS, the less change in delta DSC. There was no significant correlation between delta VAS and the delta modularity with *r* = 0.201 and *p* = 0.226 for *rest* and *r* = 0.183 and *p* = 0.285 for *DSC*, indicating no linear correlation between the difference in network measures and behavior.

## Discussion

The debate about fatigue has led to the question whether induced fatigue in healthy individuals (i.e., increased sensation of fatigue after effort) is comparable with pathological fatigue. This scenario would imply that pathological fatigue is only a more intense form of fatigue compared to what healthy individuals experience. It would also suggest a common underlying neural mechanism, as discussed by Wylie et al. (2020). In a second scenario, pathological fatigue would be seen as qualitatively different from induced fatigue in healthy individuals (which is both time-limited and alleviated by rest), having a different underlying pathophysiological mechanism and, consequently, should not be viewed as an extreme on one single fatigue continuum, as discussed by Rönnbäck and Johansson (2022). Under the first assumption, we would expect some common brain network configuration to be impacted, leading to fatigue (e.g., a similar change in network properties). This change would occur regardless of whether the fatigue was induced or pathological.

In the present study, we tried to bring some clarity to this question by evaluating network modularity since it is a common measure of the network topology which has been identified to be associated with both pathological fatigue (Messé et al., 2013) and induced sensation of fatigue by fatigability in healthy adults (Ben Simon et al., 2017). We let healthy adults and individuals suffering from pathological fatigue after mild TBI perform on a 2.5 h long, cognitively intense test battery.

Our results indicate that brain networks of patients with pathological fatigue do not display more segregation or integration compared to healthy controls, nor do we find network properties that differ depending on whether patients with pathological fatigue perform a task compared to periods of rest. When looking over both timepoints, patients and controls have a comparable level of modularity for rest and task, and no significant difference is detected between the groups (see Figure 3). These results contradict (Messé et al., 2013), which reported lower modularity for individuals with post-concussion symptoms. On the other hand, the results from Gay et al. (2016) and Høgestøl et al. (2019), that increased fatigue in chronic fatigue and MS patients is associated with less connectivity in the frontal cortex, are in line with our result. However, since we have used previously analyzed data and had no specific hypothesis about how the network properties would vary between conditions, these results should be considered exploratory. Our results highlight features that can guide hypothesis in future confirmatory studies relating to both network analyses using fNIRS and key issues in the experimental design when studying chronic fatigue.

As for our third question: are the brain networks of patients with pathological fatigue and healthy control differently affected by prolonged cognitive activity? The answer is yes; the patients’ modularity increased for both rest and task due to the prolonged activity, while the modularity stayed the same for the controls. If we compare this to the behavioral data presented in Skau et al. (2019), both groups reported increased state fatigue, and no group performed worse on the second task—the controls improved their performance. In contrast, patients performed similarly in the first task (see Figures 2B,C). Together this means that the prolonged cognitive activity increased state fatigue since both groups reported increased state fatigue (higher values on the VAS post-experiment). However, the controls did not display any change in modularity, whereas the patients did, suggesting that the fatigue in patients has a different underlying neural or network correlate. This increase in segregation after prolonged mental activity for patients with pathological fatigue could be part of an explanation for the abnormally long recovery time and something future investigation needs to determine.

Changes in modularity could be instantiated in several ways by either within-community edges weakening (splitting the community into two) or by strengthening between-community edges. Further, modularity changes are identified in several related pathological conditions. In their review of the connectivity in stroke patients, Baldassarre et al. (2016) highlight that stroke patients show more network segregation and that the increased cognitive performance after recovery correlated with an increase/restoration of functional connectivity. Similarly, Fleischer and colleagues reviewed the literature on MS that has used the graph theoretical approach for network integration and found that increased modularity was not only typical for MS, but the increase in modularity also negatively correlated with cognitive ability and MS symptom progression (Fleischer et al., 2019). For Parkinson’s disease, a recent review found that the global efficiency, another graph theoretical measure, was decreased in patients compared to healthy controls (Tessitore et al., 2019). Community segregation in these patient groups is often pathophysiologically interpreted as a consequence of reorganization or adaptation to the neurological disease or acute/chronic neural inflammation. While all these issues may impact modularity, conversely, pathological fatigue is a very common symptom in these patient groups (stroke, MS, and Parkinson’s disease) (Kluger et al., 2013), and research about the overlap between fatigue symptoms and network modularity across different patient groups will be important for our understanding of pathological fatigue.

In a recent paper, Rönnbäck and Johansson (2022) proposed the theory that pathological fatigue after TBI is due to neuro-inflammation in the CNS caused by the trauma. They argue that neuro-inflammation would affect astroglial cells and their ability to fine-tune the extracellular glutamate levels and clearance of excessive glutamate from the extracellular space. The prolonged mental activity would lead to increased excitatory glutamate in the extracellular space, which would cause swelling of astrocytes and shrinkage of the extracellular space. Neural signaling would become less specific, and the shrinking of extracellular space would result in the non-specific activation of adjacent neurons (Rönnbäck and Johansson, 2022). Our results would support such a hypothesis since a latent neuro-inflammatory process could, after prolonged activity, result in diffuse neuronal signaling, causing segregation of functional networks. For controls, assuming the absence of neuro-inflammation, neuronal signaling would not become diffused after prolonged activity; consequently, no functional network segregation would be detected. This interpretation would also support the second scenario mentioned above, that there is no true continuum between the fatigue of healthy adults and the fatigue experienced by individuals suffering from pathological fatigue. However, the leap from cellular events to large-scale network activity is currently not warranted due to a lack of sufficient data. It should be seen as a working hypothesis until more studies become available.

## Limitations

Due to the study’s design, there was a time difference between the first resting state session and the first DSC, whereas the last resting state session was right after the last DSC. Digitizing the placement of the fNIRS optodes was done, but the measurements were too noisy and were concluded to be unreliable. Therefore, we do not have external measurements confirming the channel localization. However, head size measurements were taken before the experiment. EasyCap sizes 54, 56, and 58 were used to fit the participants’ heads as accurately as possible using face and 10/20 head landmarks to get measurements where intended.

Neither coffee intake during the day nor sleep quality of the previous night were controlled for, which might also impact network properties. Although the study was exploratory, the small sample size is a limitation.

Since there was no additional time point after the end of the experiment to evaluate whether the connectivity configurations recovered for the patient group, we cannot rule out that the observed Time vs. Group interaction is not driven by fluctuations unrelated to the effort. Since the recovery time for individuals with pathological fatigue is prolonged, it would be fruitful in future research to investigate several time points after a long and cognitively intense experiment to focus on the recovery of the network properties.

## Conclusion

This exploratory analysis suggests increased segregation in the frontal cortex for patients with pathological fatigue after prolonged mental activity but not for healthy controls. Future research should determine if this pattern holds in other patient groups suffering from pathological fatigue, how long and intense the mental activity needs to be to generate segregation in the frontal cortex, and how long the recovery time needs to be to reach the baseline level of modularity.

## Data availability statement

The raw data supporting the conclusions of this article will be made available by the authors, without undue reservation.

## Ethics statement

The studies involving human participants were reviewed and approved by The Regional Ethical Review Board in Gothenburg. The patients/participants provided their written informed consent to participate in this study.

## Author contributions

SS conceived, planned, and carried out the experiments, conceptualization of analysis in this manuscript, statics, and writing—first draft. BJ conceived, planned, and carried out the experiments and writing—review and editing. H-GK wrote—reviewed and edited the manuscript WT contributed to conceptualization of analysis in this manuscript, connectivity analysis, and writing—first draft. All authors contributed to the article and approved the submitted version.
