# Supplementary material for: Segregation over time in functional networks in prefrontal cortex for individuals suffering from pathological fatigue after traumatic brain injury
Source: Front Neurosci. 2022 Sep 8;16:972720. doi: 10.3389/fnins.2022.972720 (PMC9492975; doi:10.3389/fnins.2022.972720)
Supplement: Supplementary file 1 [file Data_Sheet_1.docx]

Supplementary Material

**Segregation over time in functional networks in prefrontal cortex for individuals suffering from pathological fatigue after traumatic brain injury**

**Simon Skau**^1,2*^, **Birgitta Johansson**^1^, **Hans-Georg Kuhn**^1^, **William Hedley Thompson**^3,4^

^1^ Institute of Neuroscience and Physiology, Sahlgrenska Academy, University of Gothenburg, Gothenburg, Sweden.

^2^ Department of Pedagogical, Curricular and Professional Studies, Faculty of Education, University of Gothenburg, Gothenburg, Sweden

^3^ Department of Applied Information Technology, University of Gothenburg, Gothenburg, Sweden.

^4^ Department of Clinical Neuroscience, Karolinska Institute, Stockholm, Sweden.

***Correspondence:**Simon Skau

simon.skau@gu.se

| **Supplementary Table 1. Post Hoc test for the Group vs. Time interaction** | | |  |  |  |
| --- | --- | --- | --- | --- | --- |
|  |  | **Mean Difference** | **SE** | **t** | **P_holm_** |
| TBI, First | Control, First | -0.017 | 0.017 | -0.979 | 1.000 |
|  | TBI, Last | -0.035 | 0.013 | -2.812 | 0.049 |
|  | Control, Last | -0.009 | 0.017 | -0.507 | 1.000 |
| Control, First | TBI, Last | -0.019 | 0.017 | -1.104 | 1.000 |
|  | Control, Last | 0.008 | 0.014 | 0.591 | 1.000 |
| TBI, Last | Control, Last | 0.027 | 0.017 | 1.574 | 0.604 |

Results are averaged over the levels of: Rest/Task, TBI, traumatic brain injury; SE, standard error, P_holm_, p value with Holm correction adjusted for comparing a family of 6

| 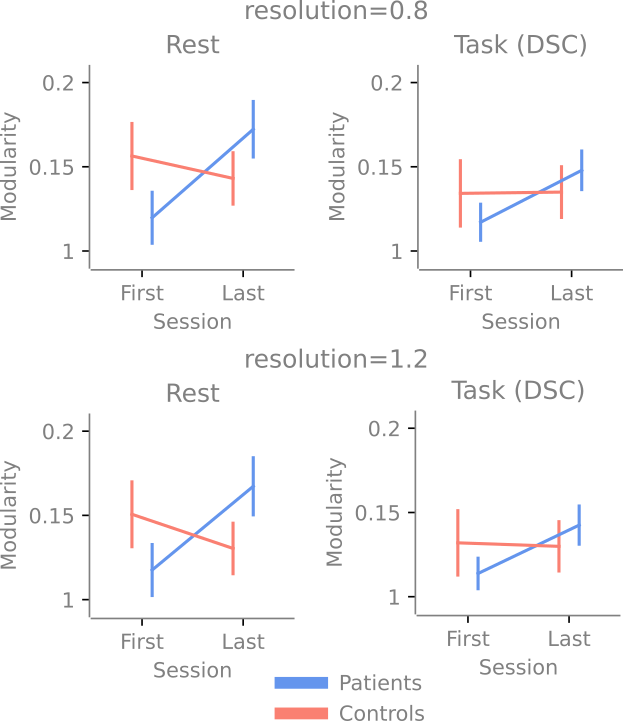 |
| --- |
| ***Supplementary Figure 1.*** *The same as figure 3, aside from changing the resolution parameter of the community detection algorithm.* |
